# Cancer metabolism of cone photoreceptors

**DOI:** 10.18632/oncotarget.5963

**Published:** 2015-10-04

**Authors:** Thierry Léveillard

**Affiliations:** INSERM, U968, Sorbonne Universités, UPMC Univ Paris 06, UMR_S 968, Institut de la Vision, CNRS, UMR_7210, Paris, France

**Keywords:** thioredoxin, retinal degeneration, nucleoredoxin-like-1, aerobic glycolysis

**Figure 1:**
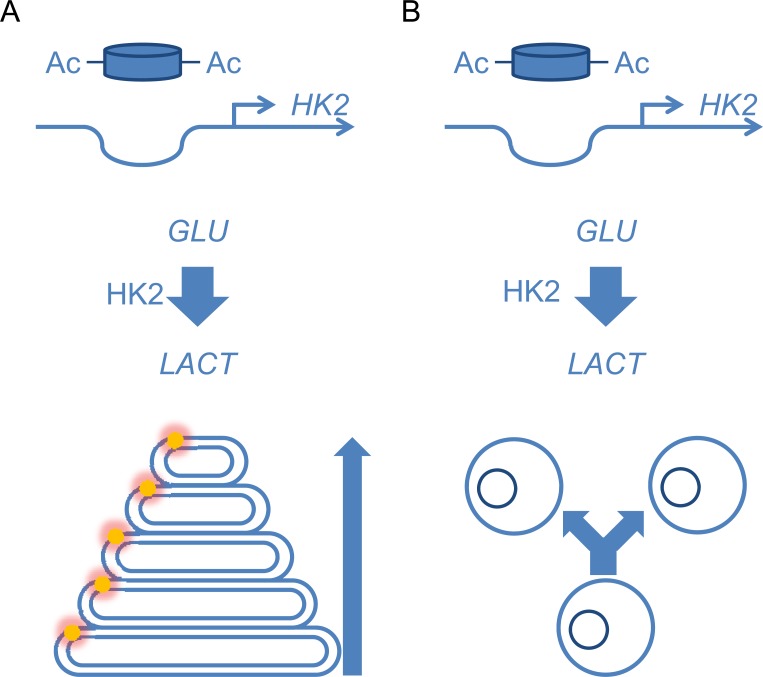
Aerobic glycolysis **A.** in cone photoreceptors. **B.** in cancer cells.

Cancer cells divert cellular physiology for their own growth through activation of proto-oncogenes and inactivation of tumor suppressors by somatic mutations, amplifications, translocations and loss of alleles. The multistep tumor progression is a succession of clonal expansions of cells with mutant genotypes that acquire traits that enable them to become tumorigenic and ultimately malignant [1]. Cancer cells generate through genomic instability random mutations and among them rare genetic changes promoting tumor formation. The reprogramming of energetic metabolism as hallmark of cancer cells was recently revisited [2]. This feature of cancer metabolism has been discovered sixty years ago by Otto Warburg who showed that most cancer cells metabolize high amounts of glucose that is secreted as lactate even in the presence of oxygen, a phenomenon named aerobic glycolysis since then [3]. This aberrant metabolism appeared originally wasteful given how little ATP is produced by glycolysis compared to oxidative phosphorylation. It produces mainly carbohydrate metabolites that are essential for cell proliferation and is compensated by a dramatic increase in glucose uptake by cancer cell. This phenomenon is used clinically to visualize tumors by 2-fluoro-6-deoxyglucose positron emission tomography. Recent results indicate that for cancer cells, the redirection of glucose toward aerobic glycolysis is not a consequence of the activation of proto-oncogene or of the inactivation of tumor suppressor genes but the first stage of multistep tumorigenesis. This is illustrated by the fact that the inactivation of the NAD-dependent protein deacetylase SIRT6 results in cell transformation by rewiring energetic metabolism to aerobic glycolysis independently of any other oncogenic event [4]. SIRT6-dependent deacetylation of histone H3 limits the expression of genes encoding glycolytic enzymes. SIRT6 deletion increase the levels of acetylated histone H3 that results in an increase expression of genes involved in aerobic glycolysis. However aerobic glycolysis is not specific to cancer cells (Figure 1B).

Vision is ensured in the retina by two different types of photoreceptors. Rods are very sensitive to light and are saturated in daylight, while cones are responsible of color vision and all visual performance during the day. The membranal opsin molecule is bound to a chromophore that isomerizes following photon capture, triggering a conformation change of opsin that is transduced into electrophysiological signal by phototransduction. The conformational change requires that opsin is embedded in a lipid bilayer of optimal fluidity, fluidity that comes from its lipid composition enriched in polyunsaturated fatty acids. Unfortunately, the degree of unsaturation of fatty acids is proportional to their tendency to oxidize. Oxidized lipids are removed daily through phagocytosis by the retinal pigmented epithelium. Opsins are embedded into lipid bilayers that are stacked in membranal discs which form the outer segment of the photoreceptor. It is the morphology of this segment that distinguishes the rods from the cones. This segment is not positioned toward the incident light as one could think intuitively but toward the retinal pigmented epithelium to allow its phagocytosis. To ensure continuity of the system, the outer segments are renewed at the same pace of 10% of their length per day. Photoreceptors are high-performance light sensors but consume at lot of energy provided by underlying choroidal circulation, which has the highest blood flow of the entire organism. We developed a therapeutic strategy aimed at preventing vision loss in patients suffering from retinitis pigmentosa, the most common form of inherited retinal degeneration. Our approach is based on the fact that rods secret a trophic factor, rod derived cone viability factor (RdCVF) that is necessary for cone survival [5]. Globally, the 57 mutations identified to date that cause individually retinitis pigmentosa trigger death of rods which is irremediably followed by that of cones due to the loss of RdCVF, expressed specifically by rods. RdCVF is an alternative splice product of the *NXNL1* gene that binds basigin-1 (BSG1), its cell surface receptor on cones [6]. BSG1 forms a complex with the glucose transporter GLUT1 at the cone surface whose transport activity is increased by RdCVF. Glucose is metabolized by cones via aerobic glycolysis to produce metabolites necessary for renewing cone outer segments (Figure 1A). Cone survival relies on the ability of RdCVF to stimulate aerobic glycolysis.

One could speculate that this observation explains why human retinoblastomas derive only from cones even if cones represent only 5% of photoreceptors [7]. Photoreceptor metabolism is giving us a unique opportunity to address unresolved questions related to the role of aerobic glycolysis. For example, we have observed that the expression of genes involved in aerobic glycolysis, among which hexokinase 2 is increased in parallel to the post-natal maturation of photoreceptors of the mouse retina [6]. Does this profile result from a progressive histone acetylation at the *Hk2* locus? This kind of epigenetic regulation would provide long term maintenance of aerobic glycolysis, presumably necessary for ensuring the daily renewal of cone outer segments during life (Figure 1A). For cancer cells, it is difficult to determine whether mitochondrial dysfunctions are causing aerobic glycolysis or whether increased glycolytic flux occurring first suppresses oxidative phosphorylation by the Crabtree effect. In the cones, aerobic glycolysis is certainly not established at the expense of oxidative phosphorylation since cone function relies on ATP even more than that of rods. Finally, if aerobic glycolysis by cancer cells is not a consequence but the cause of tumorigenesis, one could identify using functional genomics approaches, signaling pathways that differentiate cancer cells from cones, the later staying locked in an undividing stage while metabolizing glucose through aerobic glycolysis.
